# Travel-Associated *Salmonella mbandaka* Sacroiliac Osteomyelitis in a Healthy Adolescent

**DOI:** 10.1155/2013/543147

**Published:** 2013-03-27

**Authors:** Kirsty Le Doare, Elspeth Brooker, Shamez Ladhani

**Affiliations:** Paediatric Infectious Diseases Research Group, St. George's Hospital, Blackshaw Road, London SW17 0TE, UK

## Abstract

Pyogenic infections of the sacroiliac joint are rare and usually caused by *Staphylococcus aureus*. We describe a case of a 16 year-old gymnast who was subsequently diagnosed with *Salmonella mbandaka* sacroiliac osteomyelitis with adjacent psoas abscess and hepatitis one week after returning from a holiday in Crete. This case highlights a rare presentation of a common travel-associated foodborne infection.

## 1. Introduction

Pyogenic sacroiliitis is uncommon in children, representing only 1%-2% of all cases of septic arthritis, and is usually caused by *Staphylococcus aureus* [1]. Initial symptoms are often nonspecific and difficult to differentiate from septic arthritis of the hip. Diagnosis of pyogenic sacroiliitis is difficult because of its deep location and may be delayed due to the lack of specific clinical signs, which in turn may lead to complications such as sequestration or abscess formation, prolonged period of sepsis, and long-term joint deformity [2]. Here, we describe an extremely rare case of *Salmonella mbandaka *sacroiliitis with psoas abscess, osteomyelitis, and hepatitis in an adolescent gymnast presenting one week after returning from a holiday in Crete, along with a review of the published literature on previously reported cases.

## 2. Case Report

A previously healthy 16-year-old girl presented to our hospital emergency department with sudden onset of back pain and difficulty mobilising one week after returning from holiday with her twin sister to Crete. She was an avid gymnast who trained four times a week but denied any previous injuries. Clinical examination was unremarkable, and she was discharged home with a diagnosis of musculoskeletal pain. Over the next 24 hours, her pain worsened and progressed to her left buttock, radiating down the back of her leg, which prompted her family to consult a private adult neurologist. An MRI scan of her spine was performed and the neurologist diagnosed sciatica-piriformus syndrome, probably secondary to a gymnastic injury. The following day, however, she developed a high fever with rigors, but no diarrhoea or vomiting, and re-presented to our emergency department. At that time, her temperature was 40°C and she reported a 10/10 pain score. Physical examination revealed 4 cm nontender liver and 3 cm spleen. There was tenderness on palpation over both sacroiliac (SI) joints, worse on the left with pain radiating to the left upper thigh. Active and passive left hip flexion and internal/external rotation were both severely restricted because of excruciating pain. Her position of comfort was lying on her right side with her left hip flexed and externally rotated. There were no signs of enthesitis. 

Her white blood cell (WBC) count was 7.4 × 10^9^/L (neutrophils, 4.2 × 10^9^/L), haemoglobin 9.8 g/dL, platelets 286 × 10^9^/L, alanine aminotransferase 66 U/L, gamma glutamyl-transferase 275 U/L, creatine kinase 46 U/L, erythrocyte sedimentation rate >120 mm/h, and C-reactive protein 121 mg/L. Her urinalysis was normal. A radiograph of the spine and pelvis was normal while her abdominal ultrasound showed mild diffuse periportal echogenic changes in an enlarged liver with a marginally enlarged spleen. She was admitted to the paediatric ward and started on intravenous ceftriaxone along with regular analgesia. 

**Figure 1:**
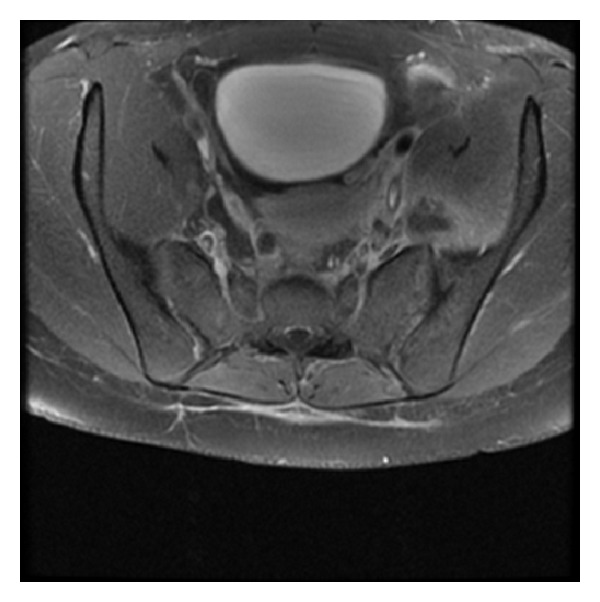
MRI of SI joints demonstrating an area of focal enhancement in the left sacroiliac joint and a septated abscess in the left iliopsoas muscle.

The next day, a bone scan demonstrated osteomyelitis involving the left SI joint and an MRI scan revealed an abnormality in the left SI joint, and the left iliac bone adjacent to the SI joint where a focal area of peripheral rim enhancement with central nonenhancement measuring 1 cm in diameter (thought to represent a bony sequestrum) was visualised, together with a complex septated abscess within the left iliopsoas muscle. Because of their location and small size, the lesions were not easily accessible for radiologically guided aspiration (Figure 1).

Forty-eight hours after admission, her blood cultures grew a fully sensitive *Salmonella* sp. subsequently identified as *S. mbadanka*. Upon requestioning the patient, she recalled that she had eaten salad leaves (including alfalfa sprouts) while on holiday and that her sister had had a short-lasting vomiting illness at that time. Over the course of the next few days, the patient's condition improved gradually. After completion of 14 days of intravenous ceftriaxone, she was discharged home with a plan to complete 6 weeks of oral ciprofloxacin and weekly clinical review in the paediatric ward. The patient's liver function tests, erythrocyte sedimentation rate, and C-reactive protein normalized within 3 weeks. An MRI of her spine performed 6 weeks after hospital discharge demonstrated persistence of the changes in the left sacroiliac joint and adjacent bone but resolution of the psoas abscess. Three months after hospital discharge, the patient was pain free with complete resolution of her symptoms and full mobility and had returned to her routine gymnastic training. 

## 3. Discussion

Bacterial sacroiliitis is an uncommon infection of the spine that occurs most commonly in children and young adults and is thought to arise from haematogenous spread of the bacteria [3]. Pyogenic sacroiliac joint infections account for ~1.5% of childhood suppurative arthritis cases [2]. Around 10% of children with pyogenic sacroiliitis have a history of pelvic trauma, which may also be the case in our patient who was an avid gymnast. Diagnosis of osteoarticular infections in the pelvic region is challenging because of the lack of specific symptoms or signs. The onset of disease is insidious in two-thirds of cases [4], and the typical symptoms of fever, buttock pain, and limping gait are often absent, especially in young children [3]. Furthermore, because of the complex anatomy of the sacroiliac joint, the pain is commonly referred to other sites such as the lower back, abdomen, thigh, or hip and can mimic a number of processes other than sacroiliitis [3]. Clinical examination may be helpful; palpation of sacroiliac joint, the FABER test (flexion, forced abduction, and external rotation) of the ipsilateral hip, and hyperextension of the hip (Gaenslen's test) may provide a clue to the diagnosis [5]. Although these provocative tests have proven to be reliable in terms of sensitivity, specificity, and predictive values in determining the source of pain in many chronic conditions, they are often not performed in children because a diagnosis of pyogenic sacroiliitis is rarely considered even after obtaining a thorough medical history. Most cases of pyogenic sacroiliitis are unilateral, with the right sacroiliac joint more frequently involved than left side, although bilateral involvement is not uncommon [3]. 

Radiological imaging plays a critical role in confirming the diagnosis. Initially, plain radiographs are often normal, but widening of the joint space of the affected side followed by blurring of subchondral bone and demineralization appears later in the course of the infection [5]. MRI is the imaging technique with the highest sensitivity and specificity (95% and 100%, resp.) for confirming pyogenic sacroiliitis and for monitoring treatment [3]. MRI combines good visualisation of the complicated anatomy of the sacroiliac joint with the ability to localise different degrees of inflammation and oedema, in the sacroiliac joint, bone marrow, and soft tissues [6, 7]. In sacroiliitis with local abscess formation, MRI can detect spinal involvement which may subsequently require surgical intervention. Although bone scans are both sensitive and specific for sacroiliitis, there is little evidence that they are superior to MRI and should be reserved for suspected cases where MRI scans appear normal [4].

Identification of the organism is important to ensure administration of appropriate antibiotic therapy. Blood cultures are positive in around half the cases [3], while synovial fluid cultures have higher yield, although the procedures for obtaining such samples are more invasive. Sacroiliac joint synovial fluid aspiration, in particular, is technically difficult because the joint is deep seated and relatively inaccessible [8]. This invasive procedure is, therefore, not warranted routinely [3] but should be considered in patients with clinical and radiographic features suggestive of pyogenic sacroiliitis who have repeated negative blood cultures and/or are not responding to empiric intravenous antibiotics [4]. 

The most common bacterial pathogen recovered from blood/synovial fluid is *Staphylococcus aureus*, accounting for 80% of pyogenic sacroiliitis in children. *Salmonella* sp. is a rare cause of pyogenic sacroiliitis. However, a recent review of 23 pediatric cases of *Salmonella* sacroiliitis reported in the English literature found the infection to be more prevalent in female adolescents who typically presented with back pain and fever that, once recognized, had a favorable therapeutic outcome [9]. Since this review, one further case report of *S. enteritidis* sacroiliitis, notably with hepatitis as in our case, was identified (Table 1).

*S. mbandaka* is a serovar of *S. enterica*, named after Mbadanka in the Democratic Republic of Congo, where it was first identified. It is classified as one of the nontyphoidal *Salmonellae* (NTS) and is usually associated with self-limiting gastroenteritis. It is a rare but increasingly reported cause of gastroenteritis outbreaks associated with alfalfa sprouts in California (2007) and Sweden (2010) and with poultry in Poland (2001) and Ireland (2008). Approximately 23 *S. mbadanka* cases are reported in the UK annually (HPA data, 2006). In developed countries, *Salmonella* infections are mainly identified in returning travelers and there are concerns regarding multidrug resistant *Salmonella* in many parts of the world [10]. Eliciting a history of recent travel and identification of the organism are, therefore, vital in order to guide empiric antimicrobial therapy. 

In summary, pyogenic sacroiliitis should always be included in the differential diagnosis of any child with fever and pain in the back, hip, buttock, or upper thigh. The FABER test should be performed routinely in such patients and, if positive, an MRI is recommended to rule out pyogenic sacroiliitis. Antibiotic therapy has proved effective in most cases with good clinical response and with no sequelae during followup. 

## Figures and Tables

**Table 1:** Case reports of *Salmonella* sacroiliitis in children since 2000.

Case	Author	Year	Country	Age (y)	Sex	Serotype
1	Penny	2012	UK	15	M	*S*. *enteritidis*, PT 6a (blood)
2	Feldman	2006	USA	14	F	*S. montevideo* (stool and synovial)
3	Kadir	2004	Kenya	14	F	*Salmonella* group D (stool and synovial)
4	Sharief	2003	Denmark	16	F	*Salmonella *group D (stool and synovial)
5	Genizi	2002	Israel	2.5	F	Virchow (blood)
6	Attarian	2001	USA	15	M	New Mexico (synovial)
